# Study protocol: baby-OSCAR trial: Outcome after Selective early treatment for Closure of patent ductus ARteriosus in preterm babies, a multicentre, masked, randomised placebo-controlled parallel group trial

**DOI:** 10.1186/s12887-021-02558-7

**Published:** 2021-02-26

**Authors:** Samir Gupta, Edmund Juszczak, Pollyanna Hardy, Nimish Subhedar, Jonathan Wyllie, Wilf Kelsall, Sunil Sinha, Sam Johnson, Tracy Roberts, Elisabeth Hutchison, Justine Pepperell, Louise Linsell, Jennifer L. Bell, Kayleigh Stanbury, Marketa Laube, Clare Edwards, David Field, on behalf of the ‘The Baby-OSCAR Collaborative Group’

**Affiliations:** 1 University Hospital of North Tees, Hardwick Road, Stockton-On-Tees, TS19 8PE UK; 2 National Perinatal Epidemiology Unit (NPEU) Clinical Trials Unit, Nuffield Department of Population Health, University of Oxford, Old Road Campus, Headington, Oxford, OX3 7LF UK; 3 Nottingham Clinical Trials Unit, School of Medicine, University of Nottingham, University Park Nottingham, Nottingham, NG7 2RD UK; 4 Institute of Applied Health Research, University of Birmingham, Birmingham, B15 2TT UK; 5 Liverpool Women’s NHS Foundation Trust, Crown Street, Liverpool, L8 7SS UK; 6 South Tees Hospitals NHS Foundation Trust, James Cook University Hospital, Middlesbrough, TS4 3BW UK; 7 NICU, Rosie Hospital, Cambridge University Hospital Foundation Trust, Cambridge, CB2 2QQ UK; 8 The University of Leicester, Department of Health Science, University Road, George Davies Centre, Leicester, LE1 7RH UK

**Keywords:** Newborn, Patent ductus arteriosus, PDA, Echocardiography, Preterm, Ibuprofen, Bronchopulmonary dysplasia

## Abstract

**Background:**

The question of whether to treat patent ductus arteriosus (PDA) early or wait until symptoms appear remains high on the research agenda for neonatal medicine. Around 7000 extremely preterm babies under 29 weeks’ gestation are born in the UK every year. In 40% of cases the PDA will fail to close spontaneously, even by 4 months of age. Untreated PDA can be associated with several serious and life-threatening short and long-term complications. Reliable data to support clinical decisions about PDA treatment are needed to prevent serious complications in high risk babies, while minimising undue exposure of infants. With the availability of routine bedside echocardiography, babies with a large PDA can be diagnosed before they become symptomatic.

**Methods:**

This is a multicentre, masked, randomised, placebo-controlled parallel group trial to determine if early-targeted treatment of a large PDA with parenteral ibuprofen in extremely preterm babies (23^+ 0^–28^+ 6^ weeks’ gestation) improves short and long-term health and economic outcomes. With parental informed consent, extremely preterm babies (born between 23^+ 0^–28^+ 6^ weeks’ gestation) admitted to tertiary neonatal units are screened using echocardiography. Babies with a large PDA on echocardiography, defined by diameter of at least 1.5 mm and unrestricted pulsatile PDA flow pattern, are randomly allocated to either ibuprofen or placebo within 72 h of birth. The primary endpoint is the composite outcome of death by 36 weeks’ postmenstrual age or moderate or severe bronchopulmonary dysplasia (BPD) at 36 weeks postmenstrual age.

**Discussion:**

Prophylactic pharmacological treatment of all preterm babies unnecessarily exposes them to potentially serious side effects of drug treatment, when their PDA may have closed spontaneously. However, delaying treatment until babies become symptomatic could result in loss of treatment benefit as irreversible damage may have already been done.

Targeted, early pharmacological treatment of PDA in asymptomatic babies has the potential to overcome the disadvantages of both prophylactic (overtreatment) and symptomatic approaches (potentially too late). This could result in improvements in the clinically important short-term clinical (mortality and moderate or severe BPD at 36 weeks’ postmenstrual age) and long-term health outcomes (moderate or severe neurodevelopment disability and respiratory morbidity) measured at 2 years corrected age.

**Trial registration:**

ISRCTN84264977. Date assigned: 15/09/2010.

**Supplementary Information:**

The online version contains supplementary material available at 10.1186/s12887-021-02558-7.

## Background

The Ductus Arteriosus (DA) is a vessel that allows blood from the right ventricle to bypass the fetal lungs to the placenta. In term babies it closes spontaneously after birth when breathing is established and is structurally closed after a few days. However, in a large number of preterm babies, the vessel does not close spontaneously resulting in a condition known as Patent Ductus Arteriosus (PDA). Around 7000 extremely preterm babies (< 29 weeks’ gestation) are born in the UK every year. In 40% the PDA will fail to close spontaneously, even by 4 months of age [1].

PDA is associated with a number of serious and life-threatening short and long-term complications. Complications of prematurity such as low blood pressure (hypotension), bleeding in the lungs (pulmonary haemorrhage) and brain (intraventricular haemorrhage (IVH)) present soon after birth. Other systemic complications such as necrotising enterocolitis (NEC) and bronchopulmonary dysplasia (BPD) present before discharge, and long-term health problems such as neurodevelopmental disability and chronic respiratory problems can be associated with PDA. The persistence of PDA has also been reported to be associated with an 8-fold increase in neonatal mortality [2, 3]. Additionally, it places a significant financial burden on the National Health Service (NHS).

Historically, clinicians who have been concerned about the symptomatic PDA or complications of prematurity have attempted to close PDA utilising medical (pharmacological) or surgical treatment. Traditionally, medical treatment is instituted as prophylactic treatment (within 24 h of birth); early symptomatic treatment (usually 3–7 days after birth) and late symptomatic treatment (after one week of age). Prophylactic pharmacological treatment of all preterm babies unnecessarily exposes a large proportion of them to the potentially serious side effects of drug treatment, when their PDA would have closed spontaneously. Symptomatic treatment on the contrary delays treatment while waiting for symptoms to appear and could result in a loss of treatment benefit as damage may have already been done.

Moreover, the practice of a conservative approach of not treating, seems to originate from uncertainty regarding the management of PDA rather than evidence favouring no intervention. This is because most studies conducted to date have involved more mature preterm babies (over 1000 g or 28 weeks’ gestation) whose PDA is more likely to close spontaneously. In addition, the studies were largely designed to assess PDA closure rates rather than assessing clinically important outcomes.

It is now suggested that large PDA on echocardiography in first 72 h (those with a diameter of ≥1.5 mm) through which blood flow is pulsatile and unrestricted are less likely to close spontaneously. Targeted early treatment of a large PDA whilst asymptomatic has the potential to overcome the disadvantages of both the prophylactic and symptomatic approaches. Although clinical detection of PDA whilst asymptomatic is challenging, it can be assessed using bedside echocardiography.

Non-steroidal anti-inflammatory drugs, especially indomethacin and ibuprofen have been widely used for the treatment of PDA. Short term efficacy of indomethacin and ibuprofen are equivalent in the treatment of PDA [4]. Ibuprofen however appears to reduce the risk of NEC and is associated with fewer clinical gastrointestinal and renal side effects compared to indomethacin; hence it is the drug of choice for this trial. Paracetamol has also been recently reported in case studies for closure of symptomatic PDA but further research needs to be done to establish its safety & effectiveness for early treatment of PDA [5].

The current literature falls short of providing substantive evidence on the management of PDA among extreme preterm babies leading to uncertainty and heterogeneity in clinical practices.

A recent cohort study reported the presence of a large PDA (defined as a PDA dimension of ≥1.5 mm) on day 3 in babies born before 28 weeks’ gestation was associated with a threefold increase in odds of death or severe morbidity compared with neonates without PDA (Odds Ratio (OR) 3.4; 95% Confidence Interval (CI) 1.1 to 11.0). Neonates with a large PDA were also reported to have increased odds of IVH (OR 4.2; 95% CI 1.3 to 14.0) and BPD (OR 3.7; 95% CI 1.0 to 14.0) compared with neonates with no PDA [6]. In preclinical trials, pharmacologic PDA closure is reported to improve alveolarisation and minimise the impaired postnatal alveolar development that is the pathologic hallmark of “new bronchopulmonary dysplasia (BPD)” [7]. An early selective treatment approach for closure of a PDA is suggested to trial its effect on BPD and mortality, which is the hypothesis of this trial.

We aim to determine if the selective treatment of echocardiographically confirmed large PDA in extremely preterm babies with ibuprofen within 72 h of birth reduces the incidence of death by 36 weeks postmenstrual age, or moderate or severe BPD at 36 weeks postmenstrual age.

## Methods/design

### Aim & Design

This is a multicentre, masked, randomised, placebo-controlled parallel group trial to determine if the selective treatment of a large PDA with ibuprofen within 72 h of birth in extremely preterm babies (23^+ 0^–28^+ 6^ weeks’ gestation) reduces the incidence of death by 36 weeks postmenstrual age or moderate to severe bronchopulmonary dysplasia (BPD) at 36 weeks postmenstrual age. In addition, it assesses other secondary outcomes and health outcomes at 2 years corrected age including survival without moderate or severe neurodevelopmental disability and survival without respiratory morbidity.

An economic evaluation will be carried out from the perspective of the health service. It will take the form of a cost-effectiveness analysis presented in terms of cost per major outcome averted. The incremental cost estimate for statistically significant differences in the pre-specified outcomes in primary and subgroup analyses would be computed.

Data are collected from hospital records and recorded on trial-specific case report forms (CRFs).

The main trial was preceded by an internal pilot phase which was used to assess the suitability of trial procedures and likelihood of recruitment targets being achieved. The Trial Steering Committee (TSC) reviewed pilot data and made recommendations regarding continuation. Data collected from the internal pilot phase of the trial will be included in the final analysis. The entire trial aims to recruit a total of 730 extremely preterm babies.

All enrolled babies are followed up at 2 years of age corrected for prematurity. Further longer-term follow-up at primary school age may be considered but will require separate funding. This may be undertaken as an amendment to this trial or as a separate application depending on the circumstances at the time.

**Fig. 1:**
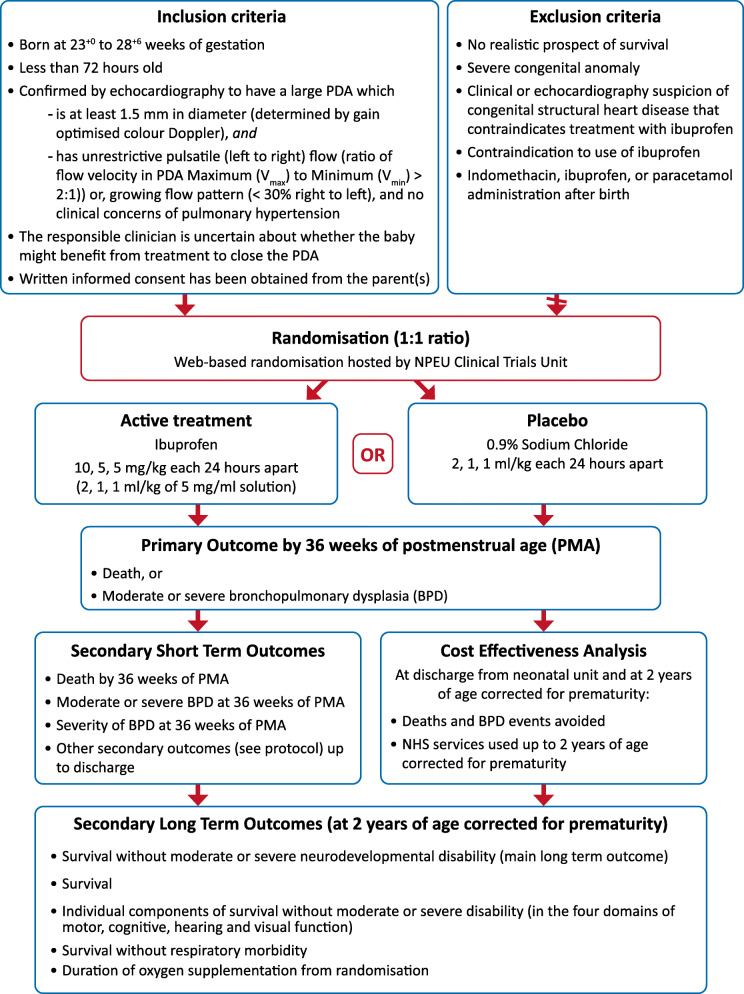
Baby-OSCAR Participant Flow Diagram

The design of the trial is summarised in Fig. 1.

### Setting & participants

The trial is recruiting in 32 neonatal units. Only units that were in equipoise for the treatment of PDA, and agreed to perform echocardiograms within 72 h of birth to confirm the presence of a large PDA were selected.

### Inclusion criteria

- Born at 23^+ 0^–28^+ 6^ weeks’ gestation
- Less than 72 h old
- Confirmed by echocardiography to have a large PDA which
  - is at least 1.5 mm in diameter (determined by gain optimised colour Doppler),
  *And*
  - has unrestrictive pulsatile (left to right) flow (ratio of flow velocity in PDA Maximum (V_max_) to Minimum (V_min_) > 2:1)) or, growing flow pattern (< 30% right to left), and no clinical concerns of pulmonary hypertension

In addition:

- The responsible clinician is uncertain about whether the baby might benefit from treatment to close the PDA
- Written informed consent is obtained from the parent(s).

### Exclusion criteria

Babies will be excluded from participation in the trial if they have:

- No realistic prospect of survival
- Severe congenital anomaly
- Clinical or echocardiography suspicion of congenital structural heart disease that contraindicates treatment with ibuprofen
- Other conditions that would contraindicate the use of ibuprofen (active bleeding especially intracranial or gastrointestinal bleeding, coagulopathy, thrombocytopenia (platelet count < 50,000), renal failure, life threatening infection, pulmonary hypertension, known or suspected necrotising enterocolitis (NEC))
- Indomethacin, ibuprofen, or paracetamol administration after birth

### Schedule of study procedures

**Table:** 

Procedure	Baby Hospitalisation					
	Screening^**1**^	Trial Entry and Treatment (days 1–3)	Up to 7 days after trial medication	3 weeks of age	36 weeks PMA^**10**^	Discharge
**Demography**^**9**^		**X**				**X**
**Echocardiogram/Colour Doppler**^**8**^	**X**			**X**		
**Confirmation of Eligibility**	**X**					
**Consent**		**X**				
**Randomisation**^**2**^		**X**				
**Ibuprofen/Placebo Dosing**^**3**^		**X**	**X**			
**IVH / PVL ultrasound scans**^**10**^			**X**		**X**	
**NEC**						**X**
**Oxygen Reduction Test**					**X**	
**SAEs**^**4**^		**X**	**X**			
**Concomitant Medication**^**5**^	**X**	**X**

**Table:** 

Infant at 2 Years Corrected Age^**6,7**^	
**Demography**^**9**^	**X**
**Visual Assessment**^**6**^	**X**
**Hearing Assessment**^**6**^	**X**
**Motor Assessment**^**6**^	**X**
**Respiratory Assessment**^**7**^	**X**
**Cognitive Assessment**^**9**^	**X**

^1^Screening assessments to be completed sufficiently in advance to enable randomisation and dosing within 72 hours of birth. If consent cannot be obtained before echocardiographic evaluation for eligibility, echocardiographic assessment should continue, and consent obtained when possible if a baby is deemed eligible.

^2^Randomisation to be completed sufficiently in advance to enable starting IMP dosing within 72 h of birth.

^3^Initial trial drug administrations to be given soon after randomisation, after 6 hours of age and within 72 hours of birth. Subsequent doses to be administered 24 hours after the initial dose.

^4^Only adverse events which are serious will be recorded from first dose until 7 days after trial medication. Only unforeseeable SAEs will be reported.

^5^Concomitant medications to be recorded only in relation to unforeseeable SAEs. In the event of an unforeseeable SAE all concomitant medication, including medication given to the baby’s mother, 7 days prior to the onset of the event to the time of its resolution must be recorded on the SAE form.

^6^Gross motor, cognitive, visual and hearing function will be assessed using the PARCA-R questionnaire, expanded to include questions to assess visual and hearing function.

^7^Respiratory assessments will be performed using a separate validated questionnaire. There will be no requirement for the infants to be assessed for respiratory and/or other neurodevelopmental functions by medically qualified personnel.

^8^An echocardiogram scan will be performed when the baby reaches around 3 weeks of age (range of 18–24 days) or at hospital discharge if discharged earlier.

^9^Demography and medications at 2 years will be assessed through the PARCA-R and other questionnaires.

^10^If a baby transfers from the recruiting site to a continuing care site for on-going care details of any scan would be helpful.

### Primary outcome

The primary outcome is defined as a composite outcome of incidence of death by 36 weeks postmenstrual age, or moderate or severe BPD at 36 weeks postmenstrual age shown in Table 1.

**Table 1:** Severity-Based Diagnostic Criteria for BPD

Time point of assessment:	36 weeks postmenstrual age
Therapy with oxygen > 21% and/or respiratory support for ≥ 28 days and the following:	
Mild BPD;	Baby is breathing room air
Moderate BPD;	Baby is in 22–29% oxygen, or 0.01–1.0 L/min
Severe BPD;	FiO_2_ ≥ 0.3, or low flow oxygen ≥ 1.1 L/min, or the baby is receiving any respiratory support (ventilation, CPAP, or high flow oxygen therapy) to achieve saturations of ≥ 91%

The need for oxygen is subjective and hence oxygen dependency is confirmed using an ‘oxygen reduction test’. This is based on the threshold at which the baby is able to maintain oxygen saturations ≥91% whilst breathing in air or at a given minimum FiO_2_. Babies unable to achieve this are considered to be oxygen dependent. This test only applies to those babies whose oxygen requirements are < 0.3, or low flow oxygen < 1.1 L/min, and who have not received any additional respiratory support in the previous 24 h. Babies outside of this are not tested, but data on their oxygen requirements will be collected.

### Secondary outcomes

#### Short term outcomes

- Death by 36 weeks postmenstrual age
- Moderate or severe BPD at 36 weeks postmenstrual age
- Severity of BPD at 36 weeks postmenstrual age (see table in Section 6.5)

Incidence or duration of the following up to discharge:

- Severe intraventricular haemorrhage (IVH) (grade III/IV with ventricular dilatation or intraparenchymal abnormality)
- Cystic periventricular leukomalacia (PVL)
- Non-cystic PVL
- Hydrocephalus
- Babies treated for retinopathy of prematurity (ROP)
- Significant pulmonary haemorrhage (fresh blood in endotracheal tube with increase in respiratory support)
- Treated for pulmonary hypertension with pulmonary vasodilator
- NEC definitive and/or complicated (Bell stage II and above) confirmed by radiology and/or histopathology
- NEC requiring surgery
- Gastrointestinal bleeding (leading to investigation or clinical treatment) within 7 days of the first dose of trial drug administration
- Spontaneous intestinal perforation
- Closed or non-significant PDA (< 1.5 mm) at around 3 weeks of age (range of 18–24 days), confirmed by ECHO
- PDA ≥ 1.5 mm at around 3 weeks of age (range of 18–24 days)
- Medical open-label treatment of a symptomatic PDA with a COX inhibitor
- Open-label treatment of a symptomatic PDA by surgical treatment
- Administration and duration of inotropic support
- Total duration of respiratory support
  - Invasive ventilation through an endotracheal tube
  - Non-invasive support through nasal CPAP, nasal ventilation, humidified high flow nasal cannula therapy, or low flow oxygen ≥1.1 L/min
- Discharge home on oxygen
- Duration of initial hospitalisation (birth to discharge home)
- Postnatal steroid use for chronic lung disease
- Tolerance of ibuprofen treatment within the foreseeable SAE reporting range, described in the protocol, section 9.1.4
- Weight gain: a change in z score between birth and discharge (or death if sooner)
- Head circumference: a change in head size z score between randomisation and discharge (or death if sooner)

#### Long term outcomes assessed at 2 years of age corrected for prematurity

- Survival without moderate or severe neurodevelopmental disability (main long-term outcome)
- Survival
- Individual components of survival without moderate or severe neurodevelopmental disability (in the four domains of motor, cognitive, hearing and visual function). Cognitive disability will be assessed by determining the standardised non-verbal cognitive subscale and language subscale scores obtained through the Parent Report of Cognitive Abilities–Revised (PARCA-R) [8] assessment. The PARCA-R assessment will be adapted to include questions to assess gross motor, hearing and visual function.
- Survival without respiratory morbidity. Respiratory morbidity will be assessed by the need for oxygen or respiratory support; presence of persistent cough and/or wheeze; need for regular treatment for respiratory illness; unscheduled attendances at hospital/GP; readmission to hospital for respiratory problems

- Duration of oxygen supplementation from randomisation

A cost-effectiveness analysis will be conducted of deaths and BPD events avoided and national health services used up to 2 years of age corrected for prematurity.

### Process outcomes

- Number of doses of trial medication received
- Adherence to protocol (e.g. protocol violations, incidence of non-symptomatic open-label treatment etc.)
- Study withdrawals

### Screening and recruitment

Extremely preterm babies potentially suitable for the trial are identified by the healthcare team within the neonatal unit. Information about the trial may be provided antenatally or soon after birth. Parent(s) are approached for consent after birth followed by echocardiographic evaluation for eligibility. However, if consent cannot be obtained echocardiographic assessment should continue and consent obtained when possible if a baby is deemed eligible.

The initial echocardiogram and Doppler assessment will incorporate:

- Size of the PDA and flow pattern according to standard trial methodology
- Size of the PDA will be determined at the site of maximum constriction (minimum diameter) using gain optimisation typically at the pulmonary end by determining the average of 3 separate clips
- If the size of the PDA is at least 1.5 mm, flow pattern will be determined by placing the pulse gate in the PDA while adjusting the velocity scale to its highest setting. If the shunt direction is > 1/3 duration of a cycle being right to left, then a rescan will be attempted after a few hours.

If the echocardiogram findings raise concerns about possibility or diagnosis of congenital heart disease, a referral will be made to a paediatric cardiologist as per clinician discretion.

### Echocardiograms

Echocardiograms are performed as part of the normal care of preterm babies. However, clinicians are required to perform an echocardiogram within 72 h of birth, at around 3 weeks of age (range of 18–24 days) or at discharge from the neonatal unit if discharged before this time.

A proportion of echocardiogram scans will be reviewed by a qualified clinician, who is not involved in recruiting for the trial, to assess consistency between clinicians.

#### Randomisation and blinding

Treatment allocation of ibuprofen or placebo are in a ratio of 1:1 and blinded such that the allocation will not be known to clinicians, the baby’s family or the trial outcome assessors.

The randomisation system is hosted by the NPEU Clinical Trials Unit. It is accessed by sites via a secure password protected system. If there are problems with accessing the system outside of normal working hours a 24 h emergency call service is available. The randomisation program uses a minimisation algorithm to ensure balance between the groups. These are allocated with respect to the size of the PDA, gestational age at birth, age at randomisation, sex, trial site, multiple births, mode of respiratory support at randomisation (1) invasive ventilation (by an endotracheal tube); or (2) non-invasive respiratory support through nasal CPAP, nasal ventilation, humidified high flow nasal cannula therapy, or low flow oxygen ≥1.1 L/min; or (3) receiving no mechanical ventilation, or pressure support (in room air, or low flow oxygen < 1.1 L/min, or ambient oxygen) and whether baby is receiving inotropes at the time of randomisation. Babies of multiple births are randomised individually.

If necessary, the treatment allocation code may be broken for a participant at the request of the site PI or clinician in charge of the baby.

### Description of intervention

Ibuprofen is supplied as a clear sterile solution at a concentration of 5 mg/ml in ampoules. Cartons contain four 2 ml single use ampoules. Each carton is labelled with a unique code and in compliance with the guidance given in Annexe 13 of the European Commission’s guidelines for Good Manufacturing Practice.

An initial loading dose of 10 mg/kg (2 ml/kg) of ibuprofen is administered, followed by two 5 mg/kg (1 ml/kg) doses at 24 and 48 h after the initial dose. Doses are calculated on the birth weight of the baby and preferably administered undiluted. If required, the IMP can be diluted to appropriate volume with 5% glucose or 0.9% Sodium Chloride. Each dose should be given as a short intravenous infusion over 15 min. All 3 doses should be given unless there are adverse effects necessitating stoppage. Placebo is supplied as a clear sterile solution of 0.9% Sodium Chloride for injection. Cartons are identical to those for ibuprofen. Volume of placebo withdrawn from the ampoule is calculated following the calculations for ibuprofen dosing.

Following randomisation, first dose should be administered soon after randomisation, after 6 h of age and within 72 h of birth. Detailed accountability records are maintained to document which pack of medication is allocated to which baby.

### Discontinuation of intervention and withdrawal

At all stages of the trial it is made clear to the parent(s) that they remain free to withdraw their baby without the need to provide reason or explanation. If parent(s) choose to withdraw their baby from trial participation, permission will be sought to complete data collection and use data up to the point of withdrawal.

A baby may also be withdrawn from the trial, if deemed by the Principal Investigator to be in their best interests.

### Safety reporting

An independent DMC has been established to review the study data and outcomes including safety reports of Serious Adverse Events (SAEs). The DMC will ensure the safety and wellbeing of the trial participants and make recommendations to the Trial Steering Committee (TSC) regarding continuance of the study or modification of the protocol. The TSC has ultimate responsibility for deciding whether the trial should be stopped on safety grounds.

Relationship of each adverse event to the trial medication is determined by a medically qualified individual. Causality of adverse events reported in the preterm newborn is difficult to assess since they may be related to the haemodynamic consequences of the patent ductus arteriosus as well as to direct effects of ibuprofen. In addition to this, high incidences of adverse events are foreseeable due to the nature of the patient population and the routine care/treatment. Consequently, only those adverse events identified as serious and not foreseeable will be recorded under trial safety reporting procedures. Safety reporting for each participant is monitored from first dose until 7 days after trial medication. Unforeseeable SAEs are reported to the NPEU CTU within 24 h of staff at site becoming aware of the event. SUSARs are reported to the MHRA and the approving Research Ethics Committee (REC) within 7 days, if the event results in death or is life-threatening, and within 15 days for all other SUSARs. In addition, a copy of the SAE form corresponding to the event is forwarded to the Chair of the DMC.

All foreseeable SAEs in the trial population that do not require reporting are specified in the full Protocol available to all sites (Additional file 1).

### Definition of end of study

The end of the trial is defined as the date when the trial database is locked. An end of trial declaration will be made to the MHRA and REC. Guidelines for early cessation of the trial have been agreed with the DMC and documented in the DMC Charter.

### Data collection

The outcome data for this trial are routinely recorded clinical items obtained from the clinical notes. No additional blood or tissue samples are required for this trial. All data is collected using trial specific CRFs. Outcome data is collected until discharge home and at 2 years corrected age.

### Project Management

The trial is sponsored by the University of Oxford and is run by the NPEU CTU, based at the University of Oxford and the Chief Investigator. On a day-to day basis, the trial is supervised by a Project Management Group (PMG) according to NPEU CTU SOPs and is subject to audit and inspection. The core PMG meets every month, either remotely or face-to-face. An extended PMG (Co-Investigator Group) meets regularly to troubleshoot, review progress and forward plan. The PMG reports to the TSC.

The trial is overseen by the TSC which has ultimate responsibility for considering and, as appropriate, acting on the recommendations of the DMC. The TSC includes an independent chair, at least one clinician, statistician and Patient and Public Involvement (PPI) representative, and the Chief Investigator. The TSC meets annually to review the progress of the trial.

The DMC is independent of the study and the TSC. The DMC reviews the progress of the trial and interim analysis at least annually and makes recommendations on the conduct of the trial to the TSC.

#### Patient and public involvement

Patient and public representatives have been extensively involved in trial planning, grant/protocol writing, and preparing study materials. Advice from two PPI co-applicants included aspects of protocol design, wording in parent-facing documentation, as well as ongoing recruitment initiatives. Contributions on documentation were also received from Bliss baby charity [9]. A lay person is a member of the TSC and is involved in trial oversight and dissemination of findings.

### Statistics and analysis

#### Sample size and power calculation

Evidence from the TIPP trial suggests that the risk of death or BPD in extremely low birth weight babies at 36 weeks postmenstrual age allocated placebo is 52% (95% CI 48 to 56%) [10, 11]. However, this trial investigated the effect of prophylactic treatment and included all babies weighing 500 − 999 g. More recent information using data derived from the latest report of Neonatal Survey Database from the Trent region [12] provides an approximate rate of death by or BPD at 36 weeks postmenstrual age of 53% for all babies admitted to the neonatal unit. These babies would have been treated according to clinical judgement and therefore a proportion of them would have been treated with ibuprofen. Given that the risk of death or BPD in babies with an echocardiographically confirmed large PDA is inherently higher, it is estimated that the risk in this group is 60%.

Su et al. (2008) [3] compared ibuprofen to indomethacin in babies ≤28 weeks’ gestation having a PDA who were less than 24 h old. The combined outcome of death within 30 days or BPD at 36 weeks postmenstrual age was observed to be 42% (95% CI 29 to 55%).

It is therefore expected, given that babies will be enrolled up to 72 h after birth, that the treatment group incidence of death/BPD at 36 weeks postmenstrual age will be approximately 48% in the intervention arm. This would imply an absolute risk reduction of 12% (60 to 48%) in the primary outcome of the trial for babies randomised to treatment compared to placebo, which is considered a clinically important difference.

Some babies will require open-label treatment (OLT) in either the treatment or placebo arm. As open-label treatment should be limited to symptomatic babies meeting only defined criteria, it is considered to have minimal or no effect on the primary outcome. Thus, adjustment of the sample size for open-label treatment is not considered necessary. A full list of OLT criteria are specified in the full Protocol available to all sites.

Regarding outcomes at 2 years corrected age, assuming the risk of a child dying before two years of age is 10%, questionnaires will be sent out to around 660 parents of surviving children. Assuming an attrition rate of 20% reduces the sample size to around 530. The proportion of infants surviving to 2 years without moderate or severe neurodevelopmental disability in the control group is expected to be 55% [13]. With outcome data available on a total sample size of around 600 (including deaths) the trial will have an 80% power to detect an increase in survival without moderate or severe neurodevelopmental disability of 11% from 55 to 66% and 90% to detect an increase of 13% from 55 to 68%.

#### Description of statistical methods

Babies will be analysed in the groups to which they are randomly assigned, comparing the outcome of all babies allocated to ibuprofen with all those allocated to placebo, regardless of deviation from the protocol or treatment received (referred to as the Intention to Treat (ITT) population).

Baseline characteristics and outcomes will be summarised with counts and percentages for categorical variables, means and standard deviations for normally distributed continuous variables, or median and interquartile range for other non-normally distributed continuous or time-to-event variables.

For binary outcomes, risk ratios and confidence intervals will be calculated using log binomial regression, or if a model fails to converge a Poisson regression model with a robust variance estimator will be used. Continuous outcomes will be analysed using linear regression models, with mean differences and confidence intervals presented for approximately normally distributed outcomes. Skewed continuous outcomes will be analysed using quantile regression models, with median differences and confidence intervals presented. Time-to-event outcomes will be analysed using Cox regression and hazard ratios with confidence intervals will be presented.

Analyses will be adjusted for all minimisation factors and the correlation between siblings from multiple births where possible. Both crude and adjusted effect estimates will be presented, but the primary inference will be based on the adjusted estimates.

Due to the multiple number of short-term outcomes, and correlation between some outcomes, statistical inference will be restricted to a predefined list of tested outcomes. Summary data by trial arm will be provided for all other outcomes, but statistical tests (or the calculation of confidence intervals) will not be performed.

Full details of the statistical analysis will be documented in the Statistical Analysis Plan.

## Discussion

### Permitted and non-permitted medications

All prescribed medications deemed necessary to provide adequate supportive care to the baby, are permitted at any stage during the trial period. However, open treatment with indomethacin or ibuprofen or other non-steroidal anti-inflammatory drugs (NSAIDs) should be avoided unless the criteria for open-label treatment are met.

The concomitant administration of other medication is not restricted but should be closely monitored for an interaction by the treating clinician.

### Open-label treatment

If the clinical condition of a baby warrants intervention, open-label treatment can be given to close the PDA (medical or surgical). The following criteria, however, have been devised to limit and rationalise the use of open-label treatment but it is recognised that clinicians may need to override this guidance in the best interests of the baby. Clinical responsibility for the care of the baby will remain fully with the neonatal clinical team irrespective of the trial.

1. Inability to wean on ventilator (ventilated for at least 7 days continuously) and any of: inability to wean oxygen; persistent hypotension; pulmonary haemorrhage; signs of cardiac failure

AND

2. Echocardiographic findings of a large PDA (≥ 2.0 mm with pulsatile flow)

AND

3. Echocardiographic findings of hyperdynamic circulation or ductal steal (refer to bBaby-OSCAR ECHO workbook)

### Participant confidentiality and retention of personal data

Contact details of the baby’s parent(s), as well as the baby’s name and any other identifying details is collected via CRFs. Parents of babies participating in the trial are informed of and provide consent to this.

Overall responsibility for ensuring that each participant’s information is kept confidential lies with the Sponsor. All paper documents are stored securely and kept in strict confidence in compliance with current data regulations. Data collected on the CRFs are stored in an electronic database held by the Trial Coordinating Centre in which the participant is identified only by a trial specific number.

Personal data are needed to contact parents when their children are 2 years of age, to co-ordinate follow up, and to disseminate the results of the trial to parent(s). Personal data is held securely and will not be used for any other purpose.

Following trial completion and report publication, data will be archived in a secure physical and electronic location with restricted access.

### Participant remuneration

A £15 voucher is sent to parents as an incentive to complete the 2-year follow-up questionnaire; however its completion is not dependent on receiving the voucher.

### Dissemination

The success of the trial depends on a large number of neonatal nurses, neonatologists, and parent(s). Credit for the trial findings will be given to all who have collaborated and participated in the trial including all local co-ordinators and collaborators, members of the trial committees, the Baby-OSCAR Co-ordinating Centre and trial staff. Authorship at the head of the primary results paper will take the form “[name], [name] and [name] on behalf of the ‘The Baby-OSCAR Collaborative Group’”. All contributors to the trial will be listed at the end of the main paper, with their contribution identified.

It is the intention of the Baby-OSCAR Collaborative Group to present data at national/international conferences, and three open-access peer-reviewed articles including the analysis of key outcomes. The NPEU Clinical Trials Unit will disseminate the results at conferences and coordinate press releases, website promotion, social and other media interest. Parents will be sent a summary of trial publications if they wish.

## Supplementary Information

**Additional file 1.**

**Additional file 2.**

**Additional file 3.**

## Data Availability

All data requests must be submitted to the corresponding author for consideration. Please note exclusive of data use will be retained until the publication of major outputs. Access to anonymised data may be granted following review of request.

## Abbreviations

AE: Adverse Event
AR: Adverse Reaction
BPD: Bronchopulmonary Dysplasia
CI: Confidence Interval
COX: Cyclo-oxygenase
CPAP: Continuous Positive Airway Pressure
DA: Ductus Arteriosus
DMC: Data Monitoring Committee
ECHO: Echocardiography
GP: General Practitioner
HRA: Health Research Authority
HTA: Health Technology Assessment
ICF: Informed Consent Form
ICH: International Conference on Harmonisation
IMP: Investigational Medicinal Product
IRAS: Integrated Research Application System
ITT: Intention to Treat
IVH: Intraventricular Haemorrhage
MHRA: Medicines and Healthcare products Regulatory Agency
NEC: Necrotising Enterocolitis
NHS: National Health Service
NIHR: National Institute for Health Research
NPEU CTU: National Perinatal Epidemiology Unit Clinical Trials Unit
NSAID: Non-Steroidal Anti-inflammatory Drug
OR: Odds Ratio
PARCA-R: Parent Report of Cognitive Abilities–Revised
PDA: Patent Ductus Arteriosus
PIL: Parent Information Leaflet
PMA: Postmenstrual Age
PMG: Project Management Group
PVL: Cystic Periventricular Leukomalacia
REC: Research Ethics Committee
RDS: Respiratory Distress Syndrome
ROP: Retinopathy of Prematurity
SAE: Serious Adverse Event
SAR: Serious Adverse Reaction
SUSAR: Suspected Unexpected Serious Adverse Reaction
TSC: Trial Steering Committee
