# Correction to: Study protocol: baby-OSCAR Trial: Outcome after Selective early treatment for Closure of patent ductus ARteriosus in preterm babies, a multicentre, masked, randomised placebo-controlled parallel group trial

**DOI:** 10.1186/s12887-021-02785-y

**Published:** 2021-07-27

**Authors:** Samir Gupta, Edmund Juszczak, Pollyanna Hardy, Nimish Subhedar, Jonathan Wyllie, Wilf Kelsall, Sunil Sinha, Sam Johnson, Tracy Roberts, Elisabeth Hutchison, Justine Pepperell, Louise Linsell, Jennifer L. Bell, Kayleigh Stanbury, Marketa Laube, Clare Edwards, David Field, on behalf of the ‘The Baby-OSCAR Collaborative Group’

**Affiliations:** 1 University Hospital of North Tees, Hardwick Road, Stockton-On-Tees, TS19 8PE UK; 2 National Perinatal Epidemiology Unit (NPEU) Clinical Trials Unit, Nuffield Department of Population Health, University of Oxford, Old Road, Campus, Headington, Oxford, OX3 7LF UK; 3 Nottingham Clinical Trials Unit, School of Medicine, University of Nottingham, University Park Nottingham, Nottingham, NG7 2RD UK; 4 Institute of Applied Health Research, University of Birmingham, Birmingham, B15 2TT UK; 5 Liverpool Women’s NHS Foundation Trust, Crown Street, Liverpool, L8 7SS UK; 6 South Tees Hospitals NHS Foundation Trust, James Cook University Hospital, Middlesbrough, TS4 3BW UK; 7 NICU, Rosie Hospital, Cambridge University Hospital Foundation Trust, Cambridge, CB2 2QQ UK; 8 Department of Health Science, The University of Leicester, University Road, George Davies Centre, Leicester, LE1 7RH UK

**Correction to: BMC Pediatr 21, 100 (2021)**

**https://doi.org/10.1186/s12887-021-02558-7**

Following the publication of the original article [1], it came to the authors’ attention that some text in the Open Label Treatment section deviated from the original wording of the protocol described.

The wording used in the original protocol is:

To consider open label (Rescue) treatment, both clinical AND echocardiographic criteria as described below should be met:

1. Clinical findings of inability to wean on ventilator (ventilated for at least 7 days continuously) AND inability to wean oxygen
  OR
  Persistent hypotension and/or pulmonary haemorrhage and/or signs of cardiac failure
2. Echocardiographic findings of a large PDA (PDA ≥ 2.0 mm with pulsatile flow)
  AND
  Hyperdynamic circulation and/or ductal steal (please refer to Baby-OSCAR ECHO workbook).

However, the section in question had used the following wording:

1. Inability to wean on ventilator (ventilated for at least 7 days continuously) and any of: inability to wean oxygen; persistent hypotension; pulmonary haemorrhage; signs of cardiac failure
  AND
2. Echocardiographic findings of a large PDA (≥ 2.0 mm with pulsatile flow)
  AND
3. Echocardiographic findings of hyperdynamic circulation or ductal steal (refer to Baby-OSCAR ECHO workbook).

## References

[1] Gupta S, Juszczak E, Hardy P (2021). Study protocol: baby-OSCAR trial: Outcome after Selective early treatment for Closure of patent ductus ARteriosus in preterm babies, a multicentre, masked, randomised placebo-controlled parallel group trial. BMC Pediatr.

