# Supplementary material for: Study protocol: baby-OSCAR trial: Outcome after Selective early treatment for Closure of patent ductus ARteriosus in preterm babies, a multicentre, masked, randomised placebo-controlled parallel group trial
Source: BMC Pediatr. 2021 Feb 26;21:100. doi: 10.1186/s12887-021-02558-7 (PMC7908699; doi:10.1186/s12887-021-02558-7)
Supplement: Supplementary file 1 — Additional file 1. [file 12887_2021_2558_MOESM1_ESM.pdf]

Foreseeable Serious Adverse Events

Foreseeable SAEs are those events which are foreseen in the patient population or as a result of the routine care/treatment of a patient.

The following serious adverse events are a foreseeable occurrence in this population of preterm babies and as such do not require reporting as SAEs:

- Anaemia requiring transfusion
- Clinically significant intracranial abnormality on cranial ultrasound scan – intracranial haemorrhage or white matter injury
- Coagulopathy requiring treatment
- Culture proven sepsis
- Death (unless unforeseeable in this population)
- Fluid retention
- Gastrointestinal bleeding
- Haematuria
- Haemothorax
- High blood creatinine level (defined as >100 µmol/L)
- Hyperbilirubinemia necessitating exchange transfusion
- Hyperglycaemia
- Hypoglycaemia
- Hypotension treated with inotropes
- Impaired renal function (urine output <0.5 ml/kg/hour, and or serum creatinine > 100 µmol/L)
- Low serum sodium level/hyponatremia (defined as sodium <130 mmol/L)
- Necrotising enterocolitis
- Neutropenia (defined as <1.0 mmol/L)
- Pneumothorax requiring treatment
- Pulmonary hypertension requiring treatment with pulmonary vasodilator
- Respiratory failure
- Seizures requiring treatment
- Significant pulmonary haemorrhage
- Spontaneous intestinal perforation
- Thrombocytopenia

Hypoglycaemia and hyperglycaemia are commonly encountered in preterm babies born below 29 weeks' gestation receiving neonatal intensive care. As per the product characteristics, the risk of hypo or hyperglycaemia does not increase with the use of Pedea® (IMP in Baby-OSCAR trial). Hence, we will include hypoglycaemia and hyperglycaemia as an expected SAE in the trial participants, but will not report data on its occurrence in the trial study groups while babies receive standard neonatal intensive care.

Given that the babies in the trial are extremely pre-term, and the expectation is that they will be in various stages of respiratory failure, the occurrence of respiratory failure will not be reported in relation to trial medication.
