# Supplementary material for: Study protocol: baby-OSCAR trial: Outcome after Selective early treatment for Closure of patent ductus ARteriosus in preterm babies, a multicentre, masked, randomised placebo-controlled parallel group trial
Source: BMC Pediatr. 2021 Feb 26;21:100. doi: 10.1186/s12887-021-02558-7 (PMC7908699; doi:10.1186/s12887-021-02558-7)
Supplement: Supplementary file 2 — Additional file 2. [file 12887_2021_2558_MOESM2_ESM.pdf]

Baby OSCAR

Outcome after selective early closure of PDA

Parent Information Leaflet

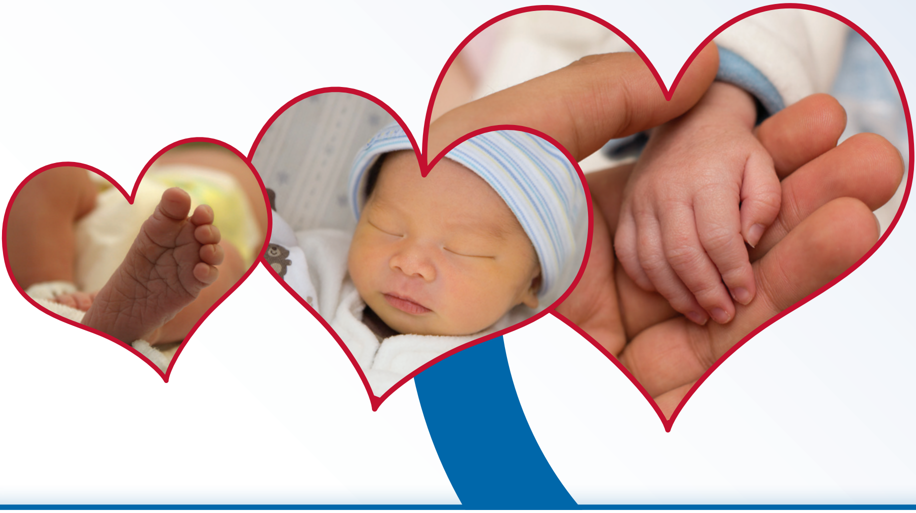

Congratulations on the birth of your baby. We appreciate this is a difficult time for you and your family and it may not seem a good moment to be talking about research. However this research is about babies, like yours, who have been born early (prematurely) so we would like to tell you about the study so you can decide whether to agree or not to your baby taking part. Before you decide we would like to explain why this study is being conducted and what it involves. Please read this information leaflet and discuss it with your friends and relatives if you wish. A member of the team caring for your baby will be happy to answer any questions that you may have.

Study title: Outcome after Selective Early Treatment for Closure of Patent Ductus ARteriosus in Preterm Babies

Short name: Baby-OSCAR

What is the purpose of this study?

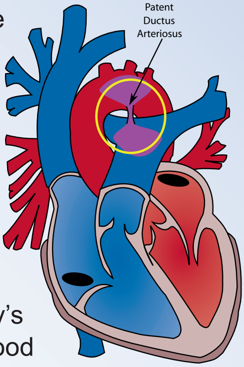

The aim of this study is to find out whether or not a large Patent Ductus Arteriosus **(PDA)** in very premature babies should be treated with ibuprofen within 72 hours of birth. Ibuprofen is a NSAID (non-steroidal anti-inflammatory drug) that is commonly used for pain relief in adults.

PDA is a condition that is caused by a blood vessel called the Ductus Arteriosus staying open after a baby's birth (patent here means open). During pregnancy, the Ductus Arteriosus allows blood from the baby's heart to flow to the mother's placenta to get oxygen, bypassing the baby's lungs. Soon after birth the ductus should close to allow blood to flow to the baby's own lungs to get oxygen. However in very premature babies the ductus often takes a long time to close on its own and this can lead to a variety of complications. Doctors are unsure if early treatment should be offered to very premature babies to close the PDA and reduce the risks of complications, or whether it would be better to wait and see (the conservative approach) if the ductus will close on its own.

PDA can be treated with medicines such as ibuprofen. However, treatment with ibuprofen can itself cause problems because premature babies may not be able to cope with its side effects. In addition, the PDA may not cause any problems. Doctors are therefore unsure whether to treat with ibuprofen as a precaution or to wait until symptoms develop, by which time some harm may have already been done.

With advances in technology doctors can now diagnose a PDA in the first 2-3 days (before it causes symptoms or signs) using a special ultrasound scan called an echocardiogram (ECHO). Using an ECHO, babies who have a large PDA (one that may not close on its own) can be identified and offered early treatment. This study aims to see whether selecting and treating these babies early is better than waiting to see if the duct closes by itself.

Why have I been invited to take part?

Parents of very premature babies (born between 23 and less than 29 weeks of pregnancy), who are less than 72 hours old and confirmed with an ECHO to have a large PDA are invited to take part in this study.

About 730 babies from neonatal units throughout the UK will be entered into the study. Initially, the study will start in a few neonatal units and later be rolled out across the country. It is anticipated that it will take about 3 years in total to complete the study. Your baby's care team will be happy to provide further information regarding the stage of the study.

Does my baby have to take part?

No. It is entirely your decision as to whether or not to allow your baby to take part in the study. If you decide not to allow your baby to take part, your baby's care will not be affected and will follow the standard approach in this unit. If you have decided initially that you would like your baby to take part, you can change your mind at any time and withdraw your baby from the study without giving a reason.

What will happen if my baby does take part?

If you agree to your baby taking part, you will be asked to sign a consent form.

Your baby's details will then be entered into a computer programme that will randomly select whether your baby will receive treatment with ibuprofen or placebo (a harmless solution of weak salt water, known as saline). This means your baby will have a 50% chance of receiving ibuprofen and a 50% chance of receiving a placebo. Neither you nor the staff caring for your baby will know which treatment has been allocated. This is to make sure the treatments are compared fairly and to prevent the results of the study being biased.

The first dose of study medication (ibuprofen or placebo) will be given after your baby is 6 hours old and within 72 hours of birth. This will be followed by two further doses 24 and 48 hours later. Each dose of ibuprofen or placebo will be given by intravenous infusion lasting 15 minutes. Your baby will be monitored closely and if it becomes clear in the days ahead that your baby needs medicines or an operation to close the PDA, your doctor will discuss this with you and the necessary treatment will be offered according to this unit's standard practice.

The tests that allow us to decide whether it is better to treat premature babies with a large PDA before symptoms appear are all part of the normal care provided in neonatal units. ECHOs may be performed at any time during your baby's treatment but one will be done in the first 72 hours and when your baby is 3 weeks old (or just before they are discharged or transferred to another hospital). The ECHO scan at 3 weeks will allow your baby's doctor to see if your baby's PDA has closed. An assessment will be done to establish whether your baby still needs additional oxygen when he/she reaches what would have been the 36th week of pregnancy, or at the time of their discharge from the unit.

Once your baby leaves hospital there will be no other tests or assessments associated with the study until your child is 2 years old based on their due date. At this time, we will send you a questionnaire that will ask how your child is developing. This is a really important part of the study. Some babies who are born early can have problems later as they grow and develop. Before you sign the consent form, please think about whether you are happy to give us this information in two years' time.

What are the possible benefits of my baby taking part?

At present we don't know whether it is better to give ibuprofen early to treat a large PDA. The early selective treatment utilises giving ibuprofen to babies with a large PDA in the first 72 hours, whereas the conservative approach utilises not treating the PDA but managing it conservatively.

We are carrying out this study to find out which approach is better to help doctors make the right decisions about the care of preterm babies in the future.

What are the possible disadvantages and risks of my baby taking part?

It is known that problems can occur in very premature babies either from having a large PDA, or by giving ibuprofen to close a PDA.

It is not known whether early selective treatment with ibuprofen of very premature babies who have a large PDA is better than a conservative management policy.

If a PDA doesn't close, it can produce a strain on the heart causing fluid to build-up in the lungs, making breathing very difficult. It can also affect how well the other organs of the body work.

However, like all medicines, ibuprofen has important side effects including problems with blood clotting and with the amount of blood flowing to the kidneys and the intestine. Therefore, giving ibuprofen can itself affect how well the body's organs function.

Ibuprofen and placebo will be given by intravenous (IV) infusion using a standard intravenous line. Almost all preterm babies admitted to intensive care need an intravenous infusion line in the first few days of life, so one will very likely be in place. Complications associated with the intravenous route of drug administration are well known and are the same for ibuprofen as for other drugs.

The staff on the Neonatal Unit monitor all babies very closely. If your baby is unwell or seems to be suffering from side effects of treatment, the doctor in charge of the care of your baby will decide whether your baby should receive further doses of ibuprofen or placebo and what the best course of action is for their care. Your baby's care will always be put before their involvement in the study.

Will taking part in this study be kept confidential?

If you take part in the study some personal information such as your name, address and other contact details will be collected. These details will be sent to the study organisers based in Oxford and will be held securely and only be used by authorised individuals to contact you when your baby reaches two years of age (based on their due date). The NHS Health and Social Care Information Centre (or a named derivative) will also be used to keep in touch with you. This service will let us know about your baby's health status. Due to the nature of neonatal research the National Perinatal Epidemiology Unit policy is to keep personal data for a period of no less than 25 years in order for us to follow-up on health related issues which may become relevant in the future. At all times your personal data will be held securely and will not be used for any other purpose.

For more information on how we process and protect your data, please see our privacy notice available at: www.npeu.ox.ac.uk/trials/privacy-notice

All other information collected for the study will not identify you or your baby. The data will be held securely and will only be accessed by authorised individuals such as the study sponsor and organisers in Oxford, the host trust and representatives from the Medicines and Healthcare products Regulatory Agency (MHRA) who ensure that studies such as this are carried out safely and correctly. Anonymised data may be shared with other researchers doing similar work.

What if relevant new information becomes available?

Sometimes we get new information about the treatment or procedure being studied. If this happens, any new information that is likely to affect your baby or their participation in the study will be discussed with you. You will be free to decide whether to continue with the study. If you decide to withdraw, the care that your baby receives will not be affected.

If the study is stopped for any reason, we will tell you and let you know what will happen next.

What will happen if I do not want my baby to continue with the study?

You can withdraw your baby from the study at any time without affecting your baby's care. We will ask you if we may use the clinical data collected up to the point of your baby's withdrawal in the analysis of the study.

What will happen to the results of the research study?

At the end of the study, the results will be analysed and published in a medical journal. We will send you a summary of the final results of the study. A copy of the full journal article can be requested from the National Perinatal Epidemiology Unit. You and your baby will not be identified in any report or publication about the study.

We may seek funding to follow up your child for the study by contacting you when they are school age. If we are successful, we will contact you through the NHS Health and Social Care Information Centre (or a named derivative).

Involvement of your General Practitioner (GP)

With your consent, your GP will be informed of your baby's participation in the study and he/she will be given a copy of this information leaflet for their records. They may then be able to help if you have queries about the study after your baby is discharged from hospital.

Who is organising and funding the research?

The study is sponsored by the University of Oxford and run by the National Perinatal Epidemiology Unit, Clinical Trials Unit and funded by the Health Technologies Assessment programme within the National Institute for Health Research [http://www.hta.ac.uk]. None of your doctors will be paid for enrolling your baby onto the study.

Who has reviewed the study?

A Research Ethics Committee will have approved all research that involves NHS patients before it goes ahead. Approval means that the Committee is satisfied that your rights will be respected, that any risks will have been reduced to a minimum and balanced against possible benefits, and that you have been given sufficient information on which to make an informed decision to take part or not. The Nottingham 2 Research Ethics Committee has reviewed and approved this study. This study has also been approved by the Medicines and Healthcare products Regulatory Authority (MHRA) which oversees the safety of new and

existing drugs. The ibuprofen formulation that will be used in this study has been approved for the treatment of PDA in premature babies and doctors have been using it for a long time.

What if there is a problem?

If at any stage you have any concerns about this study or the way it has been carried out, you should talk to the doctor or nurse who is leading the study in this hospital; their details are on the back page. Information is also available on the study website [www.npeu.ox.ac.uk/baby-oscar]

If you remain unhappy and wish to complain formally, you can do this through the NHS Complaints Procedure. Details of this procedure can be obtained from the following website http://www.nhs.uk/choiceintheNHS/Rightsandpledges/complaints/Pages/NHScomplaints.aspx. The contact telephone number for the Patient Advice Liaison Service (PALS) at this hospital is provided on the back page.

Alternatively you may contact the University of Oxford Clinical Trials and Research Governance (CTRG) office on 01865 572224 or the head of CTRG, email ctrg@admin.ox.ac.uk.

In the unlikely event that something does go wrong and your baby is harmed during the research, you may have grounds for legal action to obtain compensation against Oxford University as sponsors of the study but you may have to pay your legal costs. The NHS Complaints Procedure (mentioned above) will still be available to you.

Further information and contact details

Thank you for reading this leaflet. If you have any questions please discuss this study with the doctor or nurse who is looking after your baby. If you would like to contact an independent organisation to discuss the inclusion of babies in research studies generally we suggest that you contact Bliss, a special care baby charity. Bliss contact details are:

Bliss
for babies born premature or sick

Chapter House, 18-20 Crucifix Lane, London SE1 3JW

Free phone Family Support Helpline: 0500 618 140

Website: www.bliss.org.uk

Local contacts

Principal Investigator

{_LEAD_}

Lead Research Nurse

{_RESEARCH NURSE_}

Address

{_ADDRESS_}

{_SERVICE NAME_}

{_PALS_}

Contact address:

Baby-OSCAR

NPEU Clinical Trials Unit
National Perinatal Epidemiology Unit
University of Oxford, Old Road Campus
Headington, OXFORD. OX3 7LF

01865 617965 baby-oscar@npeu.ox.ac.uk
www.npeu.ox.ac.uk/baby-oscar

Sponsor

University of Oxford,
Clinical Trials and Research Governance (CTRG)
01865 572224 ctrg@admin.ox.ac.uk

Baby-OSCAR is funded by the National Institute for Health Research HTA Programme (project reference 11/92/15)
