# Supplementary material for: Study protocol: baby-OSCAR trial: Outcome after Selective early treatment for Closure of patent ductus ARteriosus in preterm babies, a multicentre, masked, randomised placebo-controlled parallel group trial
Source: BMC Pediatr. 2021 Feb 26;21:100. doi: 10.1186/s12887-021-02558-7 (PMC7908699; doi:10.1186/s12887-021-02558-7)
Supplement: Supplementary file 3 — Additional file 3. [file 12887_2021_2558_MOESM3_ESM.pdf]

Consent Form

Please complete in black ballpoint pen

Hospital Name: ______________________

Name of Principal Investigator: ______________________

Baby's Name: ______________________ **Study Number:** ☐☐☐☐☐

To be completed by parent(s) / guardian:

		Please initial box
1.	I confirm that I have read and understood the Parent Information Leaflet (version 7.0, dated 10 July 2018) for the above study. I have had the opportunity to consider the information, ask questions and have had these answered satisfactorily.	☐
2.	I understand that my baby's participation is voluntary and that I am free to withdraw my baby at any time before publication of the findings without giving a reason and without my baby's medical care or my legal rights being affected.	☐
3.	I understand that relevant sections of medical records and data collected during the study, relating to mother and baby, may be looked at by individuals from the sponsor, the Medicines and Healthcare products Regulatory Agency (MHRA), the co-ordinating centre or the host Trust where this is relevant to this study. I give permission for these individuals to have access to these records.	☐
4.	I agree that personal identifiable information will be collected, stored and used by the co-ordinating centre in Oxford to enable follow-up of my baby and retained as explained in the parent information leaflet.	☐
5.	I understand that information held and maintained by the Health and Social Care Information Centre and other central UK NHS bodies may be used in order to help contact me or provide information about my baby's health status.	☐
6.	I agree to my GP being informed of my baby's participation in the study.	☐
7.	I agree to my baby taking part in the study.	☐

Name of parent/guardian (*in BLOCK CAPTIALS*)

Signature

Relationship to baby

Day / Month / Year

Name of person taking consent (*in BLOCK CAPTIALS*)

Signature

Day / Month / Year

1 copy for parent(s),1 copy for Researcher's Site File,1 copy to be kept in the baby's medical notes, original for the NPEU CTU.

Baby-OSCAR is funded by the National Institute for Health Research HTA Programme (project reference 11/92/15)
